# Barriers to cardiac rehabilitation and their association with hospital readmission in patients with heart failure

**DOI:** 10.31744/einstein_journal/2025AO0713

**Published:** 2025-03-10

**Authors:** Ana Carla Soares Mota de Carvalho, Raphaela Vilar Groehs, Carolina Pereira, Vivian Lavor Soares, Tarsila Perez Mota, Sherry L Grace, Luciana Diniz Nagem Janot de Matos

**Affiliations:** 1 Hospital Israelita Albert Einstein São Paulo SP Brazil Hospital Israelita Albert Einstein, São Paulo, SP, Brazil.; 2 Escola de Enfermagem Anna Nery Rio de Janeiro RJ Brazil Escola de Enfermagem Anna Nery, Rio de Janeiro, RJ, Brazil.; 3 York University Toronto Canada York University, Toronto, Canada.; 4 KITE-Toronto Rehabilitation Institute Peter Munk Cardiac Centre University of Toronto Canada KITE-Toronto Rehabilitation Institute & Peter Munk Cardiac Centre, University Health Network,University of Toronto, Canada.

**Keywords:** Heart failure, Coronary disease, Cardiac rehabilitation, Exercise, Exercise therapy, Hospitalization, Patient discharge, Self-management, Cardiac Rehabilitation Barriers Scale, Surveys and questionnaires

## Abstract

**Objective:**

This study assessed the barriers to cardiac rehabilitation in inpatients with heart failure, the use of formal exercise supervision, and its relationship to readmissions.

**Methods:**

This study was a prospective, observational design. The Cardiac Rehabilitation Barriers Scale, the Readiness Scale focusing on physical activity, and the International Physical Activity Questionnaire were administered before hospital discharge. Participants were followed up via telephone at 30- and 90-days post-discharge, during which the International Physical Activity Questionnaire was readministered, and formal exercise supervision and readmission rates were assessed.

**Results:**

Of the 95 patients who provided consent, 88.4% were inactive. A total of 85 (89.5%) were retained at the 30-day follow-up, and 86 (90.5%) patients at the 90-day follow-up; 2 patients died. The mean total Cardiac Rehabilitation Barriers Scale score was 2.3±6.5 (out of 5), with the highest item scores for lack of energy, prior exercise, lack of awareness, distance, and exercise-related pain/fatigue. Only one participant was enrolled in cardiac rehabilitation. Nearly half had engaged in professional exercise (n=48, 56.5% at 30 days and n=45, 52.3% at 90 days) at both follow-ups. At 30 days, 25.8% of the patients were readmitted, and 25.5% were readmitted at 90 days. Participants who received professional exercise supervision within 30 days had significantly fewer readmissions (n=7, 14.6%) than those who did not (n=13, 35.1%; p=0.03).

**Conclusion:**

Barriers to cardiac rehabilitation are high among patients with heart failure. Despite access to professional exercise training, most participants remain insufficiently active. Systematic referral for cardiac rehabilitation and advocacy for coverage could mitigate poor self-management and, ultimately, reduce high readmission rates.

**Registry of Clinical Trials:**

NCT03385837

## In Brief

High rehospitalization rates and limited access to cardiac rehabilitation characterize heart failure in South America. This study highlights the significant barriers faced by patients, including lack of energy, awareness, and accessibility. Despite these challenges, professional exercise supervision has reduced readmission rates by more than 50%, underscoring its importance.

## Highlights

■ Barriers to rehabilitation: high inactivity rates (88.4%) and significant barriers, such as fatigue and lack of awareness, hinder recovery.

■ Professional supervision: only 1% of patients were enrolled in formal cardiac rehabilitation; however, those who received professional supervision experienced lower readmission rates (14.6% versus 35.1%).

■ Systematic gaps: addressing systemic gaps, such as coverage and referral to rehabilitation programs, is critical for improving patient outcomes and reducing rehospitalization rates.

## Graphical abstract

**Figure:**
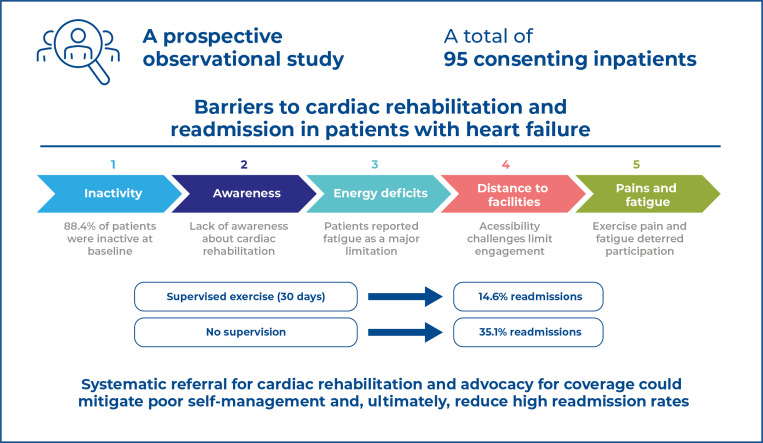

## INTRODUCTION

Heart failure (HF) is a clinical syndrome of increasing incidence, which negatively impacts patient quality of life.^1,2^ The management of HF is complex, resulting in a clinical course characterized by frequent exacerbations and costly hospitalizations.^3,4^ Mortality among patients within 90 days is approximately 10%, with a readmission rate of 25% during this period.^5,6^ This situation is true in South America, where HF is the leading cause of hospitalization, particularly in Brazil, where the mortality rate is very high.^7^

Proven strategies exist to delay and/or mitigate the high mortality and readmission rates associated with HF, primarily involving the optimization of pharmacotherapy and patient self-management.^1^ The latter includes modifications in diet, daily weight monitoring, and physical activity.^8^ Medication prescriptions and patient adherence in Brazil are often suboptimal. Furthermore, patient guidance regarding physical activity is infrequent.^7^Cardiac rehabilitation (CR) programs educate and support patients in implementing these lifestyle changes, and they have been shown to reduce mortality and morbidity, including in patients with HF.^9,10^ Structured and professionally supervised exercise is one of the main drivers of these beneficial outcomes. This is particularly important in Brazil, which has limited CR resources^11^ and where patients encounter additional barriers to effective self-management.

Cardiac rehabilitation is greatly underutilized^12^ due to barriers to participation that exist at the patient (*e.g*., cost and distance), provider (*e.g*., referral practices), and system (*e.g*., lack of programs) levels.^13^Barriers to CR have been identified in Brazil.^14-16^ However, no studies have specifically examined CR barriers in patients with HF despite their increased need for self-management and the likely greater barriers to physically accessing CR due to the physical limitations associated with the condition.

## OBJECTIVE

To identify the barriers to cardiac rehabilitation in hospitalized patients with heart failure, and to assess the engagement in cardiac rehabilitation or physiotherapy, at 30- and 90-days post-discharge and to evaluate how formal exercise engagement is related to readmission.

## METHODS

### Setting

This study was conducted at a private general hospital in São Paulo, Brazil, a middle-income country. In this institution, care is covered by private insurance purchased by many Brazilian consumers. Patients with HF generally have a length of hospital stay of 10 days and are treated in accordance with the HF management protocol, which includes a Phase I CR program. Nurses and physiotherapists educate patients and their families on the importance of physical activity, medication, daily weight monitoring, and other pertinent information. Patients were provided with medication prescriptions to be filled at their local pharmacies.

A nurse performed post-discharge monitoring via telephone. Patients are followed up by their specialist and generalist physicians as needed, their physicians who recommend CR, which patients pay for out-of-pocket.

Insufficient access to CR is available in the city; however, there is some publicly funded CR beyond the center where the study was conducted. At the center under study, a CR program was available to some patients with private insurance, but most patients had to pay out-of-pocket. The CR program is suitable for new patients and includes both exercise and education components. Patients accessed physiotherapy privately, either paid through insurance or out-of-pocket. This can occur at home or at a cardiac rehabilitation center. The number of sessions depends on the patient’s clinical status, but generally, two to three sessions per week are offered for a minimum of four months.

### Design and procedure

The study was conducted in accordance with the Declaration of Helsinki and received approval from the Research Ethics Committee of *Hospital Israelita Albert Einstein* (CAAE: 41731715.0.0000.0071; #2.515.323). All participants provided written informed consent before participating in the study.

Initial assessments were performed using validated questionnaires and investigator-generated items through face-to-face interviews conducted by a health professional on the research team. Patients were approached when clinically stable, without the administration of vasoactive drugs, and were out of the intensive care unit (during weekdays only). Patients were contacted by telephone 30 and 90 days after discharge. Recruitment of patients occurred throughout 2018 and 2019, and follow-ups were completed by February 2020, before the COVID-19 pandemic impacted care.

### Participants

Patients aged over 18 years who were hospitalized for the treatment of clinical cardiac decompensation due to systolic HF were eligible. The selected patients had a left ventricular ejection fraction (LVEF) of 50% or less. Patients hospitalized for more than 30 days, those with osteoarticular comorbidities that prohibited physical exercise, or those with cognitive issues (as assessed by the Mini-Mental State Examination [MMSE] score^17^), or who did not reside in their own homes were excluded. Additionally, patients with delirium, ischemic stroke, or visual or psychiatric conditions that prevented understanding of the questionnaires were excluded.

### Measures

The sociodemographic and clinical characteristics of the patients, including their medications, were extracted from hospital charts. Patients were asked about their exercise history as adults (yes/no).

### Cardiac Rehabilitation Barriers Scale

The Cardiac Rehabilitation Barriers Scale (CRBS), which has been translated and validated for the Brazilian population,^14^ was administered in hospital to assess barriers to enrollment and adherence to CR.^14^ It consists of 22 items, 21 of which are divided into five factors, each one representing a group of barriers: Factor 1, comorbidities/functional status (seven items); Factor 2, perceived needs (five items); Factor 3, personal/family problems (three items); Factor 4, travel/work conflicts (two items); and Factor 5, access (four items). Item 22 of the CRBS is open-ended and inquiries about other reason(s) that prevent participation in the program.^14^ Items are rated on a 5-point Likert scale ranging from 1 = Strongly Disagree to 5 = Strongly Agree.

### Readiness Scale

Based on DiClemente and Prochaska’s Stages of Change, the Readiness Scale categorizes the patient’s motivational stage concerning physical activity behavior.^18^ It consists of two questions, each scored from 1 to 10. Scores of 1 to 2 correspond to pre-contemplation (not prepared), 3 to 5 to contemplation, 6 to 8 correspond to preparation (prepared), and scores of 9 to 10 correspond to action (changing).

### International Physical Activity Questionnaire (IPAQ)

A short version of the International Physical Activity Questionnaire (IPAQ) was used to measure the level of physical activity before hospitalization.^19^ It consists of seven questions related to the frequency (days/week) and duration (time/day) of physical activities, including walking and moderate to vigorous physical efforts, as well as sedentary behavior. The level of physical activity was classified as active (IPAQ categories 1 and 2) or inactive (IPAQ categories 3 and 4), with the latter indicating that participants did not meet the minimum public health recommendations for physical activity.^19^

### Follow-up at 30 and 90 days post-discharge

All patients were contacted by telephone at 30 and 90 days following hospital discharge. The level of physical activity was assessed through the re-administration of the IPAQ-short telephone version^20^ at each call. Participants were asked whether they were undertaking supervised exercise with a physiotherapist, physical educator, or personal trainer or if they had enrolled in CR. Additionally, at both calls, participants were asked (yes/no for both) whether they weighed themselves daily and whether they required assistance with their medication (*e.g*., a family member or professional support worker hired by the family to assist elderly patients).

Finally, participants were asked if they had been hospitalized again, and if so, the number of days hospitalized and whether the cause was cardiac-related. All information obtained during the follow-up was self-reported.

### Statistical analysis

Statistical analyses were performed using SPSS v28, with the significance level set at 5%.^21,22^ Quantitative variables were described using means and standard deviations or medians and quartiles, depending on the distribution of the variables, as assessed through histograms and normality tests.^23^The association between professional exercise supervision and readmissions was evaluated using the test χ^2^.

## RESULTS

A total of 531 hospitalized patients with HF were treated during the recruitment period. After accounting for weekend discharges, exclusion criteria, and patient disinterest, the consent rate was 18%. The characteristics of the 95 participants, including their medication use, are represented in table 1. Approximately half of the patients reported a history of physical activity. Despite this history, the IPAQ scores indicated that most patients were inactive just before hospitalization and maintained sedentary behaviour for approximately 8h a day.

**Table 1:** Sociodemographic and clinical characteristics of study participants at the time of hospitalization

	n (%)	Median (1ºQ - 3ºQ)
Sex (Female)	17 (17.9)	
Age (years)		77 (71-82)
Education (number of years)		
4 years	4 (4.2)	
6 years	3 (3.2)	
8 years	13 (13.7)	
12 years	75 (78.9)	
BMI		27.5 (24.7-30.3)
Days hospitalized		11 (7-14)
Etiology of HF		
Idiopathic	15 (15.8)	
Ischemic	64 (67.4)	
Hypertensive	8 (8.4)	
Valve	5 (5.3)	
Other	3 (3.2)	
LVEF		0.35 (0.27-0.41)
NYHA classification		
Class I	8 (8.4)	
Class II	47 (49.5)	
Class III	33 (34.7)	
Class IV	7 (7.4)	
Comorbidities/Cardiovascular history (Yes)		
Previous acute myocardial infarction	55 (57.9)	
Systemic arterial hypertension	69 (72.6)	
Diabetes	47 (49.5)	
Medication (Yes)		
ACEI/ARA	54 (56.8)	
Beta-blockers	69 (72.6)	
Aldosterone antagonists	29 (30.5)	
Amiodarone	26 (27.4)	
Statin	65 (68.4)	
Metformin/Oral hypoglycemic	35 (36.8)	
Physical Exercise history	47 (49.5)	
IPAQ (inactive)	84 (88.4)	
Sedentary time (min/day)		480 (360-720)
Readiness Scale (for physical activity)		
Pre-contemplation and contemplation	19 (20)	
Preparation	41 (43.2)	
Action	35 (36.8)	

BMI: Body Mass Index; HF: heart failure; LVEF: left ventricular ejection fraction; NYHA: New York Heart Association; ACEI/ARA: Angiotensin-converting enzyme inhibitor/angiotensin II receptor antagonist; IPAQ: International Physical Activity Questionnaire

The scores for the cardiac rehabilitation Barriers Scale items are represented in table 2. The most significant individual barriers identified included lack of energy, the perception of already exercising sufficiently, and insufficient knowledge about CR. Additionally, distance to facilities, the perception of exercise as either tiring or painful, and a preference for managing health independently were notable barriers. Regarding the subscales, the primary barriers were related to “comorbidities/functional status” and “travel/work conflicts.” The “other” reported barriers were largely encompassed within the existing items; however, unique barriers included a lack of motivation or interest, a lack of perceived efficacy of CR, depression, reluctance to commit, a desire not to extend life, and unawareness of available programs nearby.

**Table 2:** Cardiac rehabilitation Barriers Scale Scores

Item	Mean	Standard deviation
1. …of distance (*e.g.*, not located in your area, too far to travel)	2.6	1.3
2. …of cost (*e.g.*, parking, gas)	2.1	1.0
3. …of transportation problems (*e.g.*, access to car, public transportation)	2.3	1.3
4. …of family responsibilities (*e.g.*, caregiving)	1.8	1.0
5. …I didn’t know about cardiac rehab (*e.g.*, the doctor didn’t tell me about it)	2.7	1.5
6. …I don’t need cardiac rehab (*e.g.*, feel well, heart problem treated, not serious)	2.3	1.2
7. …I already exercise at home or in my community	2.8	1.4
8. …severe weather	2.3	1.3
9. …I find exercise tiring or painful	2.6	1.3
10. …travel (*e.g.*, holidays, business, cottage)	2.5	1.3
11. …of time constraints (*e.g.*, too busy, inconvenient class time)	2.0	1.1
12. …of work responsibilities	2.2	1.2
13. …I don’t have the energy	3.0	1.4
14. …other health problems prevent me from going (specify:___________)	2.3	1.2
15. …I am too old	2.0	1.0
16. …my doctor did not feel it was necessary	2.2	1.2
17. … many people with heart problems don’t go, and they are fine	2.2	1.1
18. … I can manage my heart problem on my own	2.3	1.2
19. … I think I was referred, but the rehab program didn’t contact me	1.9	1.0
20. …it took too long to get referred and into the program	1.9	1.0
21. …I prefer to take care of my health alone, not in a group	2.6	1.4
Subscales		
1 - Comorbidity/functional status	2.4	1.0
2 - Perceived need	2.3	1.0
3 - Personal/family conflicts	2.3	1.0
4 - Travel/work conflicts	2.4	1.1
5 - Access	2.3	1.0
Median barrier item score (1ºQ-3ºQ)	2.3 (1.9-2.7)	
Total Barriers sum (1ºQ-3ºQ)	50.0 (40-56.5)	

Total CRBS scores differed significantly by change readiness stage (F=3.2, p<0.05), with significantly lower scores among participants in the action (2.13±0.49) than preparation stage (2.43±0.54; Least Significant Difference post-hoc test p=0.02).

### Follow-up at 30 and 90 days post-discharge

Eighty-five (89.5%) participants completed the telephone assessment in 30 days, and 86 (90.5%) completed it in 90 days. Two (2.1%) participants died by 30 days, and none died by 90 days.

Self-management behaviors at both assessment points are shown in table 3. Regarding specific physical activity, at 30 days, participants engaged in vigorous-intensity activity, a mean of 2.2±2.5 days per week for an average of 11.7±15.0 min per day, and in moderate-intensity activity, a mean of 1.9±2.1 days per week for an average of 27.6±27.2 min per day. Walking was infrequent. The mean number of sedentary minutes per day was approximately 450 at both the 30- and 90-day follow-ups, equating to 7.5 h per day. The number of sufficiently active participants was low but increased over time: pre-hospitalization (11.6%), at 30 days (17.6%), and 90 days (22.1%). Total CRBS scores were associated with the IPAQ category in the expected direction at 90 days (t=2.3, p=0.01) but not at 30 days.

**Table 3:** Self-reported self-management and readmissions at follow-up

	30 days (n=85)	90 days (n=86)
Supervised physical activity	48 (56.5)	45 (52.3)
Cardiac rehabilitation participation	0	1 (1.2)
IPAQ (insufficiently active)	69 (82.2)	66 (76.7)
Daily weighing	49 (57.6)	49 (57.0)
No medication assistance	65 (76.5)	72 (83.7)
Readmissions	22 (23.1)	22 (23.6)
Length of stay	12.8±25.8	15.2±20.7
Cardiac cause	12 (12.6)	11 (11.8)

n (%) or mean ± standard deviation shown.

IPAQ: International Physical Activity Questionnaire.

As shown in table 3, this low level of activity occurred despite approximately half of the participants reporting professional exercise supervision. Only one patient attended CR, but 48 (56.5%) participants received professional exercise supervision at 30 days, and 45 (52.3%) participants at the 90-day follow-up.

Readmissions are detailed in table 3. Participants who received professional exercise supervision at 30 days had significantly fewer readmissions (n=7, 14.6%) compared to those who did not (n=13, 25.1%; χ^2^= 4.9, p=0.03); however, no significant association was found at 90 days.

## DISCUSSION

This study assessed barriers to CR in a sample of patients. The assessment was conducted in the context of insufficient CR capacity, referral, and coverage. The results indicated relatively high barriers compared to other cohorts, even considering the absence of enrollees; chief among these barriers was a lack of energy, likely due to HF, and a few participants had already engaged in exercise. It is possible that these participants sought the assistance of a physiotherapist. Additionally, there was a lack of awareness regarding the benefits of CR.^24^ Despite approximately half of the patients working with a physiotherapist, around 80% remained insufficiently active 30 and 90 days post-discharge. Consistent with previous research, readmission rates were notably high, and the length of hospital stay was extended.^20^

IPAQ results indicated that patients with HF engaged in brief structured exercise sessions twice per week but exhibited minimal lifestyle activity and considerable sedentary time. This finding aligns with the low activity levels observed in HF samples^25^and emphasizes the diminishing perceptions that patients have regarding their health. Additionally, other studies have suggested that individuals tend to be less active on days when they participate in structured exercise.^26^ Furthermore, other HF self-management behaviors, such as daily weighing, were suboptimal, underscoring the necessity for CR. In this context, a comprehensive educational process and support for lifestyle changes, including medication adherence, are vital. These components play a crucial role in promoting self-care.

Patients with heart failure reported a high mean score for barriers to CR enrollment, which was even greater than that documented in other studies conducted within the same country.^13,16,25^ Most patients were in the preparation stage of the readiness scale to participate in CR, indicating that this was an opportune moment to intervene and encourage patient engagement in the program. This highlights the necessity of developing strategies to sustain patient motivation over time.^27^ Finally, CR effectively mitigated high readmission rates and reduced the length of hospital stays.^28^

With only one patient accessing CR, the implications of this study suggest that systematic CR referral is needed in this setting,^29,30^along with advocacy for CR coverage^31^to enhance capacity^32^and enable patient participation without financial hardship.^33^We propose that targeted educational interventions should be implemented during the hospitalization period to improve patients’ understanding of their disease, management strategies, and responsibilities regarding self-care.^33^ Additionally, recommending supervised exercise can significantly reduce a major and costly driver of morbidity in this population, specifically hospital readmissions.

Therefore, caution should be exercised when interpreting these results. First, regarding generalizability, the data were collected at a single center. Additionally, the patient profile may not represent the population of patients from non-private hospitals and, consequently, from diverse socioeconomic backgrounds. Considering the burden of HF on this population, the proportion of women in the sample was low. Furthermore, barriers to CR have been assessed in settings with limited CR coverage. Many other South American countries have two-tier health systems; however, the generalizability of these findings outside Brazil remains uncertain.

Second, selection bias could be a factor, as only approximately one-fifth of the patients treated during the recruitment period were approached, were eligible, and consented to participate. Furthermore, some patients were lost to follow-up due to the inability to contact them, which may have introduced certain retention bias. However, given the high retention rate, this is not considered a major concern.

This study has several potential limitations to this measurement. Readmissions and CR enrollments were not verified through medical charts. Additionally, socially desirable responses may have influenced the data; *e.g.*, participants were asked to self-report their daily weight and exercise. In addition, these assessments were global and did not account for specific factors such as missed days or medication adherence. Tobacco cessation and dietary habits were not considered in this study. Finally, due to the nature of the design, causal conclusions could not be drawn.

## CONCLUSION

We conclude that patients with heart failure encounter significant barriers to cardiac rehabilitation. Although half of the patients accessed physiotherapy due to coverage, those with heart failure were inadequately active and exhibited high levels of sedentary behavior. A systematic referral to cardiac rehabilitation is needed to ensure that patients receive formal exercise training and support for self-management of their condition. This approach could help mitigate the elevated readmission rates and prolonged lengths of stay associated with this population.
